# Sexual functioning and high sensory processing sensitivity

**DOI:** 10.1192/j.eurpsy.2021.1466

**Published:** 2021-08-13

**Authors:** H. Pereira, C. Nave

**Affiliations:** 1 Ubi, Research Centre in Sports Sciences, Health Sciences and Human Development, Covilha, Portugal; 2 Psychology And Education, University of Beira Interior, Covilha, Portugal

**Keywords:** sexual functioning, High Sensory Processing Sensitivity, Gender

## Abstract

**Introduction:**

The concept of Sensory Processing Sensitivity (SPS) was initially introduced by Aron in 1997 and involves complex processing of sensorial information and internal and external sensory stimuli, that is represented as an individual innate difference, as a temperamental property that concerns not only this deep sensorial processing but also to sharper general responsiveness to the environment. Its association with sexual functioning is still to be deeply determined.

**Objectives:**

Through this research we aim to evaluate the levels of High Sensory Processing Sensitivity (HSPS) and to what extent these are related to sexual functioning, in addition to assessing the mediating role of gender in this relationship.

**Methods:**

A total of 1,054 subjects between the ages of 18 and 80 (M age = 29.4; SD age = 11.9) participated in this study. Participants completed a demographic questionnaire, the Portuguese version of The Highly Sensitive Person Scale, and the Portuguese version of the Massachusetts General Hospital – Sexual Functioning Scale. The recruitment of the sample was internet-based.

**Results:**

showed that the Sensitivity Sensory Processing and Sexual Functioning variables are negatively correlated and that there are statistically significant differences in sexual functioning according to gender (t(df)=7.042; p=<.05), males scoring higher; and participants with lower levels of HSPS presented higher levels of sexual functioning (t(df)=3.599; p<.05). Finally, logistic regression showed that Gender is responsible for 6.2% of the total variance of sexual functioning.

**Conclusions:**

When working with highly sensitive people mental health professionals should take into account problems related to their sexual functioning in clinical practice.

